# IMPROVING ELDER HEALTH BEHAVIORS AND STUDENT LEARNING OUTCOMES WITH HOPE-BASED INTERPROFESSIONAL EDUCATION PROGRAM

**DOI:** 10.1093/geroni/igae098.3111

**Published:** 2024-12-31

**Authors:** Britteny Howell, Leslie Redmond, Amber Worthington, Najma Musa, Allexis Mahanna

**Affiliations:** University of Alaska, Anchorage, Alaska, United States; University of Manitoba, Winnipeg, Manitoba, Canada; University of Alaska, Anchorage, Alaska, United States; University of Alaska, Anchorage, Alaska, United States; University of Alaska Anchorage, Anchorage, Alaska, United States

## Abstract

Although Interprofessional Education (IPE) and research enhances learning, communication, and care of patients, it remains underutilized in higher education, demonstrating the need for extracurricular IPE opportunities. This presentation describes an interprofessional research project that brought together faculty, undergraduate, and graduate students from several health and social science disciplines to design and deliver a 15-week healthy aging program for older adults living in the urban Circumpolar North. Five faculty and one graduate research assistant led the project while eight students team-taught weekly, 1-hour sessions in the community focusing on healthy lifestyles within a framework of Persuasive Hope Theory. We will present the results of the student satisfaction survey regarding their involvement with the research as well as the participant satisfaction with the program. Students reported gains in “thinking like a scientist,” increased confidence conducting research tasks, benefits from teamwork, and greater consideration of the needs of older adults in their field of study. Older adult participants (N=39) also indicated favorable opinions of the program, responding that they were satisfied with the student-instructors, learning, application, impact, and value of the program. Despite small sample sizes, results from student and participant surveys both demonstrate that the IPE and intergenerational learning opportunities were meaningful. Additionally, results suggest that students may be more likely to consider a career working with older adults if given hands-on research experiences.

